# Existence of unique common solution to the system of non-linear integral equations via fixed point results in incomplete metric spaces

**DOI:** 10.1186/s13660-016-1286-7

**Published:** 2017-01-18

**Authors:** Mian Bahadur Zada, Muhammad Sarwar, Stojan Radenović

**Affiliations:** 1 Department of Mathematics, University of Malakand, Chakdara, Dir(L) Pakistan; 2 Faculty of Mechanical Engineering, Universitry of Belgrade, Kraljice Marije 16, Beograd, 11 120 Serbia

**Keywords:** 47H10, 54H25, common fixed point, weakly compatible maps, common $(CLR)$-property, common $(E.A)$-property, Urysohn integral equations, Volterra-Hammerstein integral equations

## Abstract

In this article, we apply common fixed point results in incomplete metric spaces to examine the existence of a unique common solution for the following systems of Urysohn integral equations and Volterra-Hammerstein integral equations, respectively:

$$u(s)=\phi_{i}(s)+ \int_{a}^{b}K_{i}\bigl(s, r,u(r)\bigr) \,dr, $$

where $s\in(a,b)\subseteq\mathbb{R}$; $u, \phi_{i}\in C((a,b),\mathbb{R}^{n})$ and $K_{i}:(a,b)\times(a,b)\times \mathbb{R}^{n}\rightarrow\mathbb{R}^{n}$, $i=1,2,\ldots,6 $ and

$$u(s)=p_{i}(s)+\lambda \int_{0}^{t}m(s, r)g_{i}\bigl(r,u(r) \bigr)\,dr+\mu \int_{0}^{\infty}n(s, r)h_{i}\bigl(r,u(r) \bigr)\,dr, $$

where $s\in(0,\infty)$, $\lambda,\mu\in\mathbb{R}$, *u*, $p_{i}$, $m(s, r)$, $n(s, r)$, $g_{i}(r,u(r))$ and $h_{i}(r,u(r))$, $i=1,2,\ldots,6$, are real-valued measurable functions both in *s* and *r* on $(0,\infty)$.

## Introduction and preliminaries

Mathematical models are very powerful and important parts of the mathematical analysis with numerous applications to real world problems. Several problems that appear in applied mathematics, physical sciences, geology, mechanics, engineering, economics, and biology generate mathematical models interpreted by functional equations, integral equations, matrix equations, and differential equations *etc.* There are multifarious and advanced methods, focusing on the existence of unique solutions to these models. To handle the existence of unique solution to such equations, one of these methods is the fixed point method; for example, refer to [1–4]. In metric fixed point theory the first remarkable result was given by Banach, usually known as the Banach contraction principle. This principle is a prominent tool for solving problems in non-linear analysis. Several mathematicians improved and extended this principle by modifying the interpretation and pattern of the metric function for instance: cone metric spaces [5], *G*-metric spaces [6], partial metric spaces [7] and fuzzy metric spaces [8] *etc.* After the proper introduction of cone metric space by Huang and Zhong [5], there was a drawback that fixed point results under rational type contractions are unsubstantial in a cone metric space as it is a vector-valued metric. Azam *et al.* [9] offered the conception of a complex-valued metric space for finding the fixed point results satisfying rational type contractive conditions.

### Definition 1.1

[9]

Let *Y* be non-empty set and $\mathbb{C_{+}}=\{c\in\mathbb{C}: c\succsim0\}$. Then the mapping $d:Y\times Y\rightarrow\mathbb{C_{+}}$ is a complex-valued metric if it satisfies the following axioms:

1. $d(c_{1},c_{2})=0 \Leftrightarrow c_{1}=c_{2}$;
2. $d(c_{1},c_{2})=d(c_{2},c_{1})$, for all $c_{1},c_{2}\in Y$;
3. $d(c_{1},c_{2})\precsim d(c_{1},c_{3})+d(c_{3},c_{2})$, for all $c_{1},c_{2},c_{3}\in Y$.

The set *Y* together with *d* is called a complex-valued metric space.

In this setting, Azam *et al.* [9] generalized the Banach contraction principle for two self-maps under rational type contraction. Inspired by the impact of a complex-valued metric space, several authors [4, 9–12] proceeded with the investigation of common fixed point results.

Many mathematicians applied fixed point methods to the existence of unique solutions to non-linear integral equations, for example, refer to [3, 4, 9, 13–16]. Particularly, Sintunavarat *et al.* [4] and Rashwan and Saleh [17] established fixed point results to find the existence of a unique common solution to a system of Urysohn integral equations. On the other hand, Pathak *et al.* [18] and Rashwan and Saleh [17] studied the existence of unique common solution to the system of Volterra-Hammerstein non-linear integral equations.

Throughout this manuscript *Y* represents a complex-valued metric space, unless otherwise specified. For two self-maps $f_{1}$ and $f_{2}$ defined on a non-empty set *Y*, $w\in Y$ is a common fixed point of $f_{1}$ and $f_{2}$ if $f_{1}w=f_{2}w=w$. To study common fixed points, Jungck [19] initiated the concept of weak compatibility of maps thus: $f_{1}$ and $f_{2}$ on *Y* are weakly compatible maps if $f_{1}f_{2}w=f_{2}f_{1}w$ whenever $f_{1}w=f_{2}w$, for some $w\in Y$. In the study of common fixed point results of weakly compatible mappings we often require the assumption of the continuity of mappings or the completeness of the underlying space. Regarding this Aamri and Moutawakil [20] relaxed these conditions by introducing the notion of the $(E.A)$-property. In 2011, the new notion of Common Limit in the Range property (for short $(CLR)$-property) was given by Sintunavarat and Kumam [21], which does not enforce the above mention conditions. Liu *et al.* [22] extended the $(E.A)$-property [20] to the common $(E.A)$-property and Imdad *et al.* [23] extended the $(CLR)$-property [21] to common $(CLR)$-property. Sarwar and Bahadur Zada [12] defined these views in the complex-valued metric space as follows.

### Definition 1.2

Let $f_{1},f_{2},f_{3},f_{4}:Y\rightarrow Y$ be four maps. If there are two sequences $\{z_{n}\}$ and $\{w_{n}\}$ in *Y*. Then the pairs $(f_{1},f_{3})$ and $(f_{2},f_{4})$ satisfy

1. the common $(E.A)$-property if
  $$\lim_{n\rightarrow\infty}f_{1}z_{n}=\lim _{n\rightarrow\infty }f_{3}z_{n}=\lim_{n\rightarrow\infty}f_{2}w_{n}= \lim_{n\rightarrow \infty}f_{4}w_{n}=t\in Y; $$
2. the common $(CLR_{f_{3}f_{4}})$-property if
  $$\lim_{n\rightarrow\infty}f_{1}z_{n}=\lim _{n\rightarrow\infty }f_{3}z_{n}=\lim_{n\rightarrow\infty}f_{2}w_{n}= \lim_{n\rightarrow \infty}f_{4}w_{n}=t\in f_{3}(Y)\cap f_{4}(Y). $$

Note that the $(E.A)$-property tolerates the condition of closeness of the range of subspaces of the involved mappings. However, the significance of the $(CLR)$-property reveals that closeness of the range of subspaces is not essential.

Sarwar and Bahadur Zada [12] established the following common fixed point results.

### Theorem 1.3

*Let* $f_{1}$, $f_{2}$, $f_{3}$, $f_{4}$, $f_{5}$, *and* $f_{6}$ *be six maps on* *Y* *such that*

1. $f_{1}(Y)\subseteq f_{4}(Y)$, $f_{1}(Y)\subseteq f_{5}(Y)$, $f_{2}(Y)\subseteq f_{3}(Y)$ *and* $f_{2}(Y)\subseteq f_{6}(Y)$;
2. *for all* $u,v\in Y$ *and* $0< k<1$,
  $$\begin{aligned} d(f_{1}u,f_{2}v) \precsim& k \biggl\{ \frac {d(f_{3}u,f_{1}u)d(f_{6}u,f_{1}u)d(f_{3}u,f_{2}v)d(f_{6}u,f_{2}v)}{1+d(f_{3}u,f_{2}v)d(f_{6}u,f_{2}v)+d(f_{4}v,f_{1}u)d(f_{5}v,f_{1}u)} \\ &{}+\frac {d(f_{4}v,f_{2}v)d(f_{5}v,f_{2}v)d(f_{4}v,f_{1}u)d(f_{5}v,f_{1}u)}{1+d(f_{3}u,f_{2}v)d(f_{6}u,f_{2}v)+d(f_{4}v,f_{1}u)d(f_{5}v,f_{1}u)} \biggr\} ; \end{aligned}$$
3. *the pairs* $(f_{1},f_{3})$, $(f_{2},f_{4})$, $(f_{1},f_{6})$, *and* $(f_{2},f_{5})$ *are weakly compatible*;
4. *either both the pairs* $(f_{1},f_{3})$ *and* $(f_{1},f_{6})$ *satisfies common* $(CLR_{f_{1}})$-*property or both the pairs* $(f_{2},f_{4})$ *and* $(f_{2},f_{5})$ *satisfies common* $(CLR_{f_{2}})$-*property*.

*Then* $f_{1}$, $f_{2}$, $f_{3}$, $f_{4}$, $f_{5}$, *and* $f_{6}$ *have a unique common fixed point in* *Y*.

### Theorem 1.4

*Let* $f_{1}$, $f_{2}$, $f_{3}$, $f_{4}$, $f_{5}$, *and* $f_{6}$ *be six maps on* *Y* *such that all the conditions of Theorem* 1.3 *except condition* $(4)$ *holds*. *In addition if either the pairs* $(f_{1},f_{3})$ *and* $(f_{1},f_{6})$ *or the pairs* $(f_{2},f_{4})$ *and* $(f_{2},f_{5})$ *satisfy the common* $(E.A)$-*property such that either* $f_{4}(Y)$ *and* $f_{5}(Y)$ *or* $f_{3}(Y)$ *and* $f_{6}(Y)$ *are closed subspaces of* *Y*, *then* $f_{1}$, $f_{2}$, $f_{3}$, $f_{4}$, $f_{5}$, *and* $f_{6}$ *have a unique common fixed point in* *Y*.

The aim of this manuscript is to study the existence of unique common solution for the systems of:

- Urysohn integral equations in complex-valued metric spaces,
- Volterra-Hammerstein integral equations in ordinary metric spaces.

## Existence of unique common solution to the systems of Urysohn integral equations

Our plan is to apply Theorem 1.3 to the existence of a unique common solution to the following system:

2.1 $$ u(s)=\phi_{i}(s)+ \int_{a}^{b}K_{i}\bigl(s, r,u(r)\bigr) \,dr, $$

where $s\in(a,b)\subseteq\mathbb{R}$; $u, \phi_{i} \in C((a,b),\mathbb{R}^{n})$ and $K_{i}:(a,b)\times(a,b)\times\mathbb{R}^{n}\rightarrow \mathbb{R}^{n}$, $i=1,2,\ldots,6$.

Let us denote

$$\Omega_{i}\bigl(u(s)\bigr)= \int_{a}^{b}K_{i}\bigl(s, r,u(r)\bigr) \,dr, $$

where $i=1,2,\ldots,6$.

Assume that the following conditions hold:

(C_1_): for $i=4,5$,
  $$\Omega_{1}u(s)+\phi_{1}(s)+\phi_{i}(s)- \Omega_{i} \bigl(\Omega_{1}u(s)+\phi _{1}(s)+ \phi_{i}(s) \bigr)=0, $$
(C_2_): for $j=3,6$,
  $$\Omega_{2}u(s)+\phi_{2}(s)+\phi_{j}(s)- \Omega_{j} \bigl(\Omega_{2}u(s)+\phi _{2}(s)+ \phi_{j}(s) \bigr)=0, $$
(C_3_): for $j=3,6$,
  $$\phi_{1}(s)+3\phi_{j}(s)+2\Omega_{j}u(s)+ \Omega_{1} \bigl(\Omega_{1}u(s)+\phi _{1}(s) \bigr)+\Omega_{j} \bigl(2u(s)-\Omega_{j}u(s)- \phi_{j}(s) \bigr) =4u(s), $$
(C_4_): for $i=4,5$,
  $$\phi_{2}(s)+3\phi_{i}(s)+2\Omega_{i}u(s) + \Omega_{2} \bigl(\Omega_{2}u(s)+\phi _{2}(s) \bigr)+ \Omega_{i} \bigl(2u(s)-\Omega_{i}u(s)-\phi_{i}(s) \bigr) =4u(s). $$

Let $Y=C((a,b),\mathbb{R}^{n})$, $a>0$ be an incomplete complex-valued metric space with metric

$$d(u,v)=\max_{s\in(a,b)}\bigl\Vert u(s)-v(s)\bigr\Vert _{\infty}\sqrt{1+a^{2}}e^{\dot {\iota}\arctan{a}},\quad \mbox{for all }u,v \in Y. $$

Define six operators $f_{1},f_{2},f_{3},f_{4},f_{5},f_{6}:Y\rightarrow Y$ by

2.2 $$ \left\{ \begin{aligned} &f_{1}u(s)= \Omega_{1}u(s)+\phi_{1}(s), \\ &f_{2}u(s)=\Omega_{2}u(s)+\phi_{2}(s), \\ &f_{3}u(s)=2u(s)-\Omega_{3}u(s)-\phi_{3}(s), \\ &f_{4}u(s)=2u(s)-\Omega_{4}u(s)-\phi_{4}(s), \\ &f_{5}u(s)=2u(s)-\Omega_{5}u(s)-\phi_{5}(s), \\ &f_{6}u(s)=2u(s)-\Omega_{6}u(s)-\phi_{6}(s). \end{aligned} \right\} $$

Now, we are in a position to formulate the existence results.

### Theorem 2.1

*Under the assumptions* (C_1_)-(C_4_) *if*

1. *there exist two sequences* $\{{z_{n}}\}$ *and* $\{{w_{n}}\}$ *in* *Y* *such that*
  2.3 $$ \lim_{n\rightarrow\infty}f_{2}{z_{n}}=\lim _{n\rightarrow\infty}f_{4}{z_{n}} =\lim _{n\rightarrow\infty}f_{2}{w_{n}}=\lim _{n\rightarrow\infty }f_{5}{w_{n}}=z\in f_{2}(Y); $$
2. *for each* $u,v\in Y$ *and* $0<\lambda<1$,
  $$\begin{aligned}& \Upsilon_{1}\sqrt{1+a^{2}}e^{\dot{\iota}\arctan{a}} \\& \quad \precsim\lambda \biggl\{ \frac{ \Upsilon_{2}\times\Upsilon_{3}\times\Upsilon_{4}\times\Upsilon _{5}+\Upsilon_{6}\times\Upsilon_{7}\times\Upsilon_{8}\times\Upsilon _{9}}{1+ (\max_{s\in(a,b)}\Upsilon_{4} )\cdot (\max_{s\in(a,b)}\Upsilon_{5} ) + (\max_{s\in(a,b)}\Upsilon_{8} )\cdot ( \max_{s\in(a,b)}\Upsilon_{9} )} \biggr\} , \end{aligned}$$
  *where*
  $$\begin{aligned}& \Upsilon_{1} =\bigl\Vert \Omega_{1}u(s)- \Omega_{2}v(s)+\phi_{1}(s)-\phi _{2}(s)\bigr\Vert _{\infty}\sqrt{1+a^{2}}e^{\dot{\iota}\arctan{a}}, \\& \Upsilon_{2} =\bigl\Vert 2u(s)-\Omega_{3}u(s)- \Omega_{1}u(s)-\phi_{1}(s)-\phi _{3}(s)\bigr\Vert _{\infty}\sqrt{1+a^{2}}e^{\dot{\iota}\arctan{a}}, \\& \Upsilon_{3} =\bigl\Vert 2u(s)-\Omega_{6}u(s)- \Omega_{1}u(s)-\phi _{1}(s)-\phi_{6}(s)\bigr\Vert _{\infty}\sqrt{1+a^{2}}e^{\dot{\iota}\arctan{a}}, \\& \Upsilon_{4} =\bigl\Vert 2u(s)-\Omega_{3}u(s)- \Omega_{2}v(s)-\phi _{2}(s)-\phi_{3}(s)\bigr\Vert _{\infty}\sqrt{1+a^{2}}e^{\dot{\iota}\arctan{a}}, \\& \Upsilon_{5} =\bigl\Vert 2u(s)-\Omega_{6}u(s)- \Omega_{2}v(s)-\phi _{2}(s)-\phi_{6}(s)\bigr\Vert _{\infty}\sqrt{1+a^{2}}e^{\dot{\iota}\arctan{a}}, \\& \Upsilon_{6} =\bigl\Vert 2v(s)-\Omega_{4}v(s)- \Omega_{2}v(s)-\phi _{2}(s)-\phi_{4}(s)\bigr\Vert _{\infty}\sqrt{1+a^{2}}e^{\dot{\iota}\arctan{a}}, \\& \Upsilon_{7} =\bigl\Vert 2v(s)-\Omega_{5}v(s)- \Omega_{2}v(s)-\phi _{2}(s)-\phi_{5}(s)\bigr\Vert _{\infty}\sqrt{1+a^{2}}e^{\dot{\iota}\arctan{a}}, \\& \Upsilon_{8} =\bigl\Vert 2v(s)-\Omega_{4}v(s)- \Omega_{1}u(s)-\phi _{1}(s)-\phi_{4}(s)\bigr\Vert _{\infty}\sqrt{1+a^{2}}e^{\dot{\iota}\arctan{a}}, \\& \Upsilon_{9} =\bigl\Vert 2v(s)-\Omega_{5}v(s)- \Omega_{1}u(s)-\phi _{1}(s)-\phi_{5}(s)\bigr\Vert _{\infty}\sqrt{1+a^{2}}e^{\dot{\iota}\arctan{a}}; \end{aligned}$$
3. $f_{1}(Y)\subseteq f_{4}(Y)$, $f_{1}(Y)\subseteq f_{5}(Y)$, $f_{2}(Y)\subseteq f_{3}(Y)$, *and* $f_{2}(Y)\subseteq f_{6}(Y)$ *such that* $(f_{1},f_{3})$, $(f_{2},f_{4})$, $(f_{2},f_{5})$, *and* $(f_{1},f_{6})$ *are weakly compatible*.

*Then the system* (2.1) *of Urysohn integral equations has a unique common solution*.

### Proof

Notice that the system (2.1) of Urysohn integral equations has a unique common solution if and only if the system (2.2) of operators has a unique common fixed point.

Now,

2.4 $$ \left\{ \begin{aligned} &d(f_{1}u,f_{2}v) =\max_{s\in(a,b)}\bigl\Vert \Omega _{1}u(s)- \Omega_{2}v(s)+\phi_{1}(s)-\phi_{2}(s)\bigr\Vert _{\infty}\sqrt {1+a^{2}}e^{\dot{\iota}\arctan{a}}, \\ &d(f_{3}u,f_{1}u) =\max_{s\in(a,b)}\bigl\Vert 2u(s)-\Omega _{3}u(s)-\Omega_{1}u(s)- \phi_{1}(s)-\phi_{3}(s)\bigr\Vert _{\infty}\sqrt {1+a^{2}}e^{\dot{\iota}\arctan{a}}, \\ &d(f_{6}u,f_{1}u) =\max_{s\in(a,b)}\bigl\Vert 2u(s)-\Omega _{6}u(s)-\Omega_{1}u(s)- \phi_{1}(s)-\phi_{6}(s)\bigr\Vert _{\infty}\sqrt {1+a^{2}}e^{\dot{\iota}\arctan{a}}, \\ &d(f_{3}u,f_{2}v) =\max_{s\in(a,b)}\bigl\Vert 2u(s)-\Omega _{3}u(s)-\Omega_{2}v(s)- \phi_{2}(s)-\phi_{3}(s)\bigr\Vert _{\infty}\sqrt {1+a^{2}}e^{\dot{\iota}\arctan{a}}, \\ &d(f_{6}u,f_{2}v) =\max_{s\in(a,b)}\bigl\Vert 2u(s)-\Omega _{6}u(s)-\Omega_{2}v(s)- \phi_{2}(s)-\phi_{6}(s)\bigr\Vert _{\infty}\sqrt {1+a^{2}}e^{\dot{\iota}\arctan{a}}, \\ &d(f_{4}v,f_{2}v) =\max_{s\in(a,b)}\bigl\Vert 2v(s)-\Omega _{4}v(s)-\Omega_{2}v(s)- \phi_{2}(s)-\phi_{4}(s)\bigr\Vert _{\infty}\sqrt {1+a^{2}}e^{\dot{\iota}\arctan{a}}, \\ &d(f_{5}v,f_{2}v) =\max_{s\in(a,b)}\bigl\Vert 2v(s)-\Omega _{5}v(s)-\Omega_{2}v(s)- \phi_{2}(s)-\phi_{5}(s)\bigr\Vert _{\infty}\sqrt {1+a^{2}}e^{\dot{\iota}\arctan{a}}, \\ &d(f_{4}v,f_{1}u) =\max_{s\in(a,b)}\bigl\Vert 2v(s)-\Omega _{4}v(s)-\Omega_{1}u(s)- \phi_{1}(s)-\phi_{4}(s)\bigr\Vert _{\infty}\sqrt {1+a^{2}}e^{\dot{\iota}\arctan{a}}, \\ &d(f_{5}v,f_{1}u) =\max_{s\in(a,b)}\bigl\Vert 2v(s)-\Omega _{5}v(s)-\Omega_{1}u(s)- \phi_{1}(s)-\phi_{5}(s)\bigr\Vert _{\infty}\sqrt {1+a^{2}}e^{\dot{\iota}\arctan{a}}. \end{aligned} \right\} $$

From condition (2) of Theorem 2.1, we have

$$\begin{aligned}& \Upsilon_{1}\sqrt{1+a^{2}}e^{\dot{\iota}\arctan{a}} \\& \quad \precsim\lambda \biggl\{ \frac{\Upsilon_{2}\times\Upsilon_{3}\times\Upsilon_{4}\times \Upsilon_{5}+\Upsilon_{6}\times\Upsilon_{7}\times\Upsilon_{8}\times \Upsilon_{9}}{1+ (\max_{s\in(a,b)}\Upsilon_{4} )\cdot (\max_{s\in(a,b)}\Upsilon_{5} ) + (\max_{s\in(a,b)}\Upsilon_{8} )\cdot (\max_{s\in(a,b)}\Upsilon_{9} )} \biggr\} , \end{aligned}$$

which implies that

$$\begin{aligned}& \max_{s\in(a,b)} \Upsilon_{1}\sqrt{1+a^{2}}e^{\dot{\iota }\arctan{a}} \\& \quad \precsim \lambda \biggl\{ \frac{ (\max_{s\in(a,b)}\Upsilon _{2} )\cdot (\max_{s\in(a,b)}\Upsilon_{3} )\cdot (\max_{s\in(a,b)}\Upsilon_{4} )\cdot (\max_{s\in(a,b)}\Upsilon_{5} )}{ 1+ (\max_{s\in(a,b)}\Upsilon_{4} )\cdot (\max_{s\in(a,b)}\Upsilon_{5} )+ (\max_{s\in(a,b)}\Upsilon_{8} )\cdot (\max_{s\in(a,b)}\Upsilon_{9} )} \\& \qquad {} +\frac{ (\max_{s\in(a,b)}\Upsilon _{6} )\cdot (\max_{s\in(a,b)}\Upsilon_{7} )\cdot (\max_{s\in(a,b)}\Upsilon_{8} )\cdot (\max_{s\in(a,b)}\Upsilon_{9} )}{ 1+ (\max_{s\in(a,b)}\Upsilon_{4} )\cdot (\max_{s\in(a,b)}\Upsilon_{5} )+ (\max_{s\in(a,b)}\Upsilon_{8} )\cdot (\max_{s\in(a,b)}\Upsilon_{9} )} \biggr\} , \end{aligned}$$

using (2.4), we obtain

$$\begin{aligned} d(f_{1}u,f_{2}v) \precsim&\lambda \biggl\{ \frac {d(f_{3}u,f_{1}u)d(f_{6}u,f_{1}u)d(f_{3}u,f_{2}v)d(f_{6}u,f_{2}v)}{1+d(f_{3}u,f_{2}v)d(f_{6}u,f_{2}v)+d(f_{4}v,f_{1}u)d(f_{5}v,f_{1}u)} \\ &{}+\frac {d(f_{4}v,f_{2}v)d(f_{5}v,f_{2}v)d(f_{4}v,f_{1}u)d(f_{5}v,f_{1}u)}{1+d(f_{3}u,f_{2}v)d(f_{6}u,f_{2}v)+d(f_{4}v,f_{1}u)d(f_{5}v,f_{1}u)} \biggr\} . \end{aligned}$$

Now, to show that $f_{1}(Y) \subseteq f_{4}(Y)$, we have

$$\begin{aligned} f_{4} \bigl(f_{1}u(s)+\phi_{4}(s) \bigr) =&2 \bigl[f_{1}u(s)+\phi_{4}(s) \bigr]-\Omega_{4} \bigl(f_{1}u(s)+\phi _{4}(s) \bigr)-\phi_{4}(s) \\ =&f_{1}u(s)+f_{1}u(s)+\phi_{4}(s)- \Omega_{4} \bigl(f_{1}u(s)+\phi _{4}(s) \bigr) \\ =&f_{1}u(s)+\Omega_{1}u(s)+\phi_{1}(s)+ \phi_{4}(s)-\Omega_{4} \bigl(\Omega_{1}u(s)+ \phi_{1}(s)+\phi_{4}(s) \bigr). \end{aligned}$$

Using (C_1_), we get $f_{4} (f_{1}u(s)+\phi_{4}(s) )= f_{1}u(s)$, which implies that $f_{1}(Y) \subseteq f_{4}(Y)$. Similarly, one can prove that $f_{1}(Y)\subseteq f_{5}(Y) $, $f_{2}(Y)\subseteq f_{3}(Y)$ and $f_{2}(Y)\subseteq f_{6}(Y)$.

Next, we need to show the weak compatibility of the pair $(f_{1},f_{3})$. For this, we have

2.5 $$\begin{aligned} \bigl\Vert f_{3}f_{1}u(s)-f_{1}f_{3}u(s) \bigr\Vert =&\bigl\Vert f_{3}\bigl(\Omega _{1}u(s)+ \phi_{1}(s)\bigr)-f_{1}\bigl(2u(s)-\Omega_{3}u(s)- \phi _{3}(s)\bigr)\bigr\Vert \\ =&\bigl\Vert 2\bigl(\Omega_{1}u(s)+\phi_{1}(s)\bigr)- \Omega_{3}\bigl(\Omega _{1}u(s)+\phi_{1}(s) \bigr)-\phi_{3}(s) \\ &{}-\Omega_{1}\bigl(2u(s)-\Omega_{3}u(s)- \phi_{3}(s)\bigr)-\phi _{1}(s)\bigr\Vert . \end{aligned}$$

If $f_{1}u(s)=f_{3}u(s)$, for $u(s)\in Y$. Then $\Omega_{1}u(s)+\phi _{1}(s)=2u(s)-\Omega_{3}u(s)-\phi_{3}(s)$, thus (2.5) becomes

$$\begin{aligned} \bigl\Vert f_{3}f_{1}u(s)-f_{1}f_{3}u(s) \bigr\Vert =&\bigl\Vert 2\bigl(2u(s)-\Omega_{3}u(s)- \phi_{3}(s)\bigr)-\Omega_{3} \bigl(2u(s)- \Omega_{3}u(s)-\phi_{3}(s)\bigr) \\ &{}-\phi_{3}(s)-\Omega_{1}\bigl(\Omega_{1}u(s)+ \phi_{1}(s)\bigr)-\phi _{1}(s)\bigr\Vert \\ =&\bigl\Vert 4u(s)-2\Omega_{3}u(s)-3\phi_{3}(s)- \Omega_{3} \bigl(2u(s)-\Omega_{3}u(s)-\phi_{3}(s) \bigr) \\ &{}-\Omega_{1}\bigl(\Omega_{1}u(s)+\phi_{1}(s) \bigr)-\phi_{1}(s)\bigr\Vert , \end{aligned}$$

with the help of (C_3_), we get $\|f_{3}f_{1}u(s)-f_{1}f_{3}u(s)\| =0$, which implies that $f_{3}f_{1}u(s)=f_{1}f_{3}u(s)$, whenever $f_{1}u(s)=f_{3}u(s)$. Thus $(f_{1},f_{3})$ is weakly compatible. In a similarly way one can easily show the weakly compatibility of the pairs $(f_{2},f_{4})$, $(f_{1},f_{6})$ and $(f_{2},f_{5})$. Also, from condition (1) of Theorem 2.1, the pairs $(f_{2},f_{4})$ and $(f_{2},f_{5})$ satisfy the common $(CLR_{f_{2}})$-property.

Thus by Theorem 2.1 we can find a unique common fixed point of $f_{1}$, $f_{2}$, $f_{3}$, $f_{4}$, $f_{5}$, and $f_{6}$ in *Y*, that is, the system (2.1) of Urysohn integral equations has a unique common solution in *Y*. □

In the next result we use the common $(E.A)$-property and the proof is simple, so we omit it.

### Theorem 2.2

*Under the assumptions* (C_1_)-(C_4_) *and the conditions* (2), (3) *of Theorem* 2.1, *if there exist two sequences* $\{{z_{n}}\}$ *and* $\{{w_{n}}\}$ *in* *Y* *such that*

2.6 $$ \lim_{n\rightarrow\infty}f_{2}{z_{n}}=\lim _{n\rightarrow\infty}f_{4}{z_{n}} =\lim _{n\rightarrow\infty}f_{2}{w_{n}}=\lim _{n\rightarrow\infty }f_{5}{w_{n}}=z, \quad \textit{for some } z\in Y, $$

*and both* $f_{4}(Y)$ *and* $f_{5}(Y)$ *are closed subspaces of* *Y*, *then the system* (2.1) *of Urysohn integral equations has a unique common solution*.

## Existence of unique common solution to the systems of Volterra-Hammerstein integral equations

In this section, we present the real-valued metric version of Theorem 1.3 and Theorem 1.4 and the proof can easily be obtained, so we omit its proof here.

### Corollary 3.1

*Let* $f_{1}$, $f_{2}$, $f_{3}$, $f_{4}$, $f_{5}$, $f_{6}$ *be six maps on a metric space* $(Z,d)$ *such that*

1. $f_{1}(Z)\subseteq f_{4}(Z)$, $f_{1}(Z)\subseteq f_{5}(Z)$, $f_{2}(Z)\subseteq f_{3}(Z)$, *and* $f_{2}(Z)\subseteq f_{6}(Z)$;
2. *for all* $u,v\in Z$ *and* $0< k<1$,
  $$\begin{aligned} d(f_{1}u,f_{2}v) \leq&\lambda \biggl\{ \frac {d(f_{3}u,f_{1}u)d(f_{6}u,f_{1}u)d(f_{3}u,f_{2}v)d(f_{6}u,f_{2}v)}{1+d(f_{3}u,f_{2}v)d(f_{6}u,f_{2}v)+d(f_{4}v,f_{1}u)d(f_{5}v,f_{1}u)} \\ &{}+\frac {d(f_{4}v,f_{2}v)d(f_{5}v,f_{2}v)d(f_{4}v,f_{1}u)d(f_{5}v,f_{1}u)}{1+d(f_{3}u,f_{2}v)d(f_{6}u,f_{2}v)+d(f_{4}v,f_{1}u)d(f_{5}v,f_{1}u)} \biggr\} ; \end{aligned}$$
3. *the pairs* $(f_{1},f_{3})$, $(f_{2},f_{4})$, $(f_{1},f_{6})$ *and* $(f_{2},f_{5})$ *are weakly compatible*;
4. *either both the pairs* $(f_{1},f_{3})$ *and* $(f_{1},f_{6})$ *satisfies common* $(CLR_{f_{1}})$-*property or both the pairs* $(f_{2},f_{4})$ *and* $(f_{2},f_{5})$ *satisfies common* $(CLR_{f_{2}})$-*property*.

*Then* $f_{1}$, $f_{2}$, $f_{3}$, $f_{4}$, $f_{5}$, *and* $f_{6}$ *have a unique common fixed point in* *Z*.

### Corollary 3.2

*Let* $f_{1}$, $f_{2}$, $f_{3}$, $f_{4}$, $f_{5}$, $f_{6}$ *be six maps on a metric space* $(Z,d)$ *such that all the conditions of corollary* 3.1 *except condition* $(4)$ *holds*. *In addition if either the pairs* $(f_{1},f_{3})$ *and* $(f_{1},f_{6})$ *or* $(f_{2},f_{4})$ *and* $(f_{2},f_{5})$ *satisfy the common* $(E.A)$-*property such that either* $f_{4}(Z)$ *and* $f_{5}(Z)$ *or* $f_{3}(Z)$ *and* $f_{6}(Z)$ *are closed subspaces of* *Z*, *then* $f_{1}$, $f_{2}$, $f_{3}$, $f_{4}$, $f_{5}$, *and* $f_{6}$ *have a unique common fixed point in* *Z*.

We apply the above results to study the existence of unique common solution to the following system (3.1) of non-linear Volterra-Hammerstein integral equations.

Let $Z=(L(0,\infty),\mathbb{R})$ be the space of real-valued measurable functions on $(0,\infty)$:

3.1 $$ u(s)=p_{i}(s)+\lambda \int_{0}^{t}m(s, r)g_{i}\bigl(r,u(r) \bigr) \,dr+\mu \int_{0}^{\infty}n(s, r)h_{i}\bigl(r,u(r) \bigr) \,dr, $$

for all $s\in(0,\infty)$, where $\lambda,\mu\in\mathbb{R}$, *u*, $p_{i}$, $m(s, r)$, $n(s, r)$, $g_{i}(r,u(r))$ and $h_{i}(r,u(r))$, $i= 1,2,\ldots,6$, are real-valued measurable functions in *s* and *r* on $(0,\infty)$.

Let us denote

$$\Delta_{i}u(s)= \int_{0}^{t}m(s, r)g_{i}\bigl(r,u(r) \bigr) \,dr $$

and

$$\nabla_{i}u(s)= \int_{0}^{\infty}n(s, r)h_{i}\bigl(r,u(r) \bigr) \,dr, $$

where $i=1,2,\ldots,6$.

Assume that

($\mathrm{C}^{*}_{1}$)
for $i=4,5$,
  $$\begin{aligned}& \Delta_{1}u(s)+\nabla_{1}u(s)+p_{1}(s)+p_{i}(s) -\Delta_{i} \bigl(\Delta _{1}u(s)+\nabla_{1}u(s)+p_{1}(s)+p_{i}(s) \bigr) \\& \quad {} -\nabla_{i} \bigl(\Delta_{1}u(s)+ \nabla_{1}u(s)+p_{1}(s)+p_{i}(s) \bigr)=0, \end{aligned}$$
($\mathrm{C}^{*}_{2}$)
for $j=3,6$,
  $$\begin{aligned}& \Delta_{2}u(s)+\nabla_{2}u(s)+p_{2}(s)+p_{j}(s) -\Delta_{j} \bigl(\Delta _{2}u(s)+\nabla_{2}u(s)+p_{2}(s)+p_{j}(s) \bigr) \\& \quad {} -\nabla_{j} \bigl(\Delta_{2}u(s)+ \nabla_{2}u(s)+p_{2}(s)+p_{j}(s) \bigr) =0, \end{aligned}$$
($\mathrm{C}^{*}_{3}$)
for $j=3,6$,
  $$\begin{aligned}& p_{1}(s)+3p_{j}(s)+2\Delta_{j}u(s)+2 \nabla_{j}u(s)+\Delta_{1} \bigl(\Delta _{1}u(s)+ \nabla_{1}u(s)+p_{1}(s) \bigr) \\& \quad {}+\Delta_{j} \bigl(2u(s)-\Delta_{j}u(s)- \nabla_{j}u(s)-p_{j}(s) \bigr)+\nabla _{1} \bigl(\Delta_{1}u(s)+\nabla_{1}u(s)+p_{1}(s) \bigr) \\& \quad {} +\nabla_{j} \bigl(2u(s)-\Delta_{j}u(s)- \nabla_{j}u(s)-p_{j}(s) \bigr) =4u(s), \end{aligned}$$
($\mathrm{C}^{*}_{4}$)
for $i=4,5$,
  $$\begin{aligned}& p_{2}(s)+3p_{i}(s)+2\Delta_{i}u(s)+2 \nabla_{i}u(s)+\Delta_{2} \bigl(\Delta _{2}u(s)+ \nabla_{2}u(s)+p_{2}(s) \bigr) \\& \quad {} +\Delta_{i} \bigl(2u(s)-\Delta_{i}u(s)- \nabla_{i}u(s)-p_{i}(s) \bigr)+\nabla _{2} \bigl(\Delta_{2}u(s)+\nabla_{2}u(s)+p_{2}(s) \bigr) \\& \quad {} +\nabla_{i} \bigl(2u(s)-\Delta_{i}u(s)- \nabla_{i}u(s)-p_{i}(s) \bigr)=4u(s). \end{aligned}$$

Let $Z=(L(0,\infty),\mathbb{R})$ be an incomplete metric space with metric

$$d(u, v)=\max_{s\in(0,\infty)}\bigl\| u(s)-v(s)\bigr\| ,\quad \text{for all } u,v\in Z . $$

Define the six operators $f_{1}$, $f_{2}$, $f_{3}$, $f_{4}$, $f_{5}$, and $f_{6}$ on *Z* by

3.2 $$ \begin{aligned} &f_{1}u(s)= \Delta_{1}u(s)+\nabla_{1}u(s)+p_{1}(s), \\ &f_{2}u(s)=\Delta_{2}u(s)+\nabla_{2}u(s)+p_{2}(s), \\ &f_{3}u(s)=2u(s)-\Delta_{3}u(s)-\nabla_{3}u(s)-p_{3}(s), \\ &f_{4}u(s)=2u(s)-\Delta_{4}u(s)-\nabla_{4}u(s)-p_{4}(s), \\ &f_{5}u(s)=2u(s)-\Delta_{5}u(s)-\nabla_{5}u(s)-p_{5}(s), \\ &f_{6}u(s)=2u(s)-\Delta_{6}u(s)-\nabla_{6}u(s)-p_{6}(s). \end{aligned} $$

Now, we are in a position to formulate the existence results.

### Theorem 3.3

*Under the assumptions* ($\mathrm{C}_{1}^{*}$)-($\mathrm{C}_{4}^{*}$), *if*

1. *there exist two sequences* $\{{z_{n}}\}$ *and* $\{{w_{n}}\}$ *in* *Z* *such that*
  3.3 $$ \lim_{n\rightarrow\infty}f_{2}{z_{n}}=\lim _{n\rightarrow\infty }f_{4}{z_{n}}=\lim _{n\rightarrow\infty}f_{2}{w_{n}}=\lim _{n\rightarrow\infty }f_{5}{w_{n}}=z\in f_{2}(Z); $$
2. *for each* $u,v\in Z$ *and* $0<\lambda<1$,
  $$\begin{aligned}& \bigl\Vert \Delta_{1}u(s)+\nabla_{1}u(s)+p_{1}(s)- \Delta_{2}u(s)-\nabla _{2}u(s)-p_{2}(s)\bigr\Vert \\& \quad \leq\lambda \biggl\{ \frac{ \Upsilon_{2}\times\Upsilon_{3}\times\Upsilon_{4}\times\Upsilon _{5}+\Upsilon_{6}\times\Upsilon_{7}\times\Upsilon_{8}\times\Upsilon _{9}}{1+ (\max_{s\in(a,b)}\Upsilon_{4} )\cdot (\max_{s\in(a,b)}\Upsilon_{5} ) + (\max_{s\in(a,b)}\Upsilon_{8} )\cdot (\max_{s\in(a,b)}\Upsilon_{9} )} \biggr\} , \end{aligned}$$
  *where*
  $$\begin{aligned}& \Upsilon_{2} =\bigl\Vert 2u(s)-\Delta_{3}u(s)- \nabla_{3}u(s)-p_{3}(s)-\Delta _{1}u(s)-\nabla _{1}u(s)-p_{1}(s)\bigr\Vert , \\& \Upsilon_{3} =\bigl\Vert 2u(s)-\Delta_{6}u(s)- \nabla_{6}u(s)-p_{6}(s)-\Delta _{1}u(s)-\nabla _{1}u(s)-p_{1}(s)\bigr\Vert , \\& \Upsilon_{4} =\bigl\Vert 2u(s)-\Delta_{3}u(s)- \nabla_{3}u(s)-p_{3}(s)-\Delta _{2}v(s)-\nabla _{2}v(s)-p_{2}(s)\bigr\Vert , \\& \Upsilon_{5} =\bigl\Vert 2u(s)-\Delta_{6}u(s)- \nabla_{6}u(s)-p_{6}(s)-\Delta _{2}v(s)-\nabla _{2}v(s)-p_{2}(s)\bigr\Vert , \\& \Upsilon_{6} =\bigl\Vert 2v(s)-\Delta_{4}v(s)- \nabla_{4}v(s)-p_{4}(s)-\Delta _{2}v(s)-\nabla _{2}v(s)-p_{2}(s)\bigr\Vert , \\& \Upsilon_{7} =\bigl\Vert 2v(s)-\Delta_{5}v(s)- \nabla_{5}v(s)-p_{5}(s)-\Delta _{2}v(s)-\nabla _{2}v(s)-p_{2}(s)\bigr\Vert , \\& \Upsilon_{8} =\bigl\Vert 2v(s)-\Delta_{4}v(s)- \nabla_{4}v(s)-p_{4}(s)-\Delta _{1}u(s)-\nabla _{1}u(s)-p_{1}(s)\bigr\Vert , \\& \Upsilon_{9} =\bigl\Vert 2v(s)-\Delta_{5}v(s)- \nabla_{5}v(s)-p_{5}(s)-\Delta _{1}u(s)-\nabla _{1}u(s)-p_{1}(s)\bigr\Vert ; \end{aligned}$$
3. $f_{1}(Z)\subseteq f_{4}(Z)$, $f_{1}(Z)\subseteq f_{5}(Z)$, $f_{2}(Z)\subseteq f_{3}(Z)$ *and* $f_{2}(Z)\subseteq f_{6}(Z)$ *such that the pairs* $(f_{1},f_{3})$, $(f_{2},f_{4})$, $(f_{1},f_{6})$ *and* $(f_{2},f_{5})$ *are weakly compatible*,

*then the system* (3.1) *of Volterra*-*Hammerstein equations has a unique common solution*.

### Proof

Notice that the system of Volterra-Hammerstein non-linear integral equations (3.1) has a unique common solution if and only if the system of operators (3.2) has a unique common fixed point.

Now,

3.4 $$ \left\{ \begin{aligned} &d(f_{1}u,f_{2}v) =\max_{s\in(0,\infty)}\bigl\Vert \Delta _{1}u(s)+ \nabla_{1}u(s)+p_{1}(s)-\Delta_{2}u(s)-\nabla _{2}u(s)-p_{2}(s)\bigr\Vert , \\ &d(f_{3}u,f_{1}u) =\max_{s\in(0,\infty)}\bigl\Vert 2u(s)-\Delta _{3}u(s)-\nabla_{3}u(s)-p_{3}(s)- \Delta_{1}u(s)-\nabla _{1}u(s)-p_{1}(s)\bigr\Vert , \\ &d(f_{6}u,f_{1}u) =\max_{s\in(0,\infty)}\bigl\Vert 2u(s)-\Delta _{6}u(s)-\nabla_{6}u(s)-p_{6}(s)- \Delta_{1}u(s)-\nabla _{1}u(s)-p_{1}(s)\bigr\Vert , \\ &d(f_{3}u,f_{2}v) =\max_{s\in(0,\infty)}\bigl\Vert 2u(s)-\Delta _{3}u(s)-\nabla_{3}u(s)-p_{3}(s)- \Delta_{2}v(s)-\nabla _{2}v(s)-p_{2}(s)\bigr\Vert , \\ &d(f_{6}u,f_{2}v) =\max_{s\in(0,\infty)}\bigl\Vert 2u(s)-\Delta _{6}u(s)-\nabla_{6}u(s)-p_{6}(s)- \Delta_{2}v(s)-\nabla _{2}v(s)-p_{2}(s)\bigr\Vert , \\ &d(f_{4}v,f_{2}v) =\max_{s\in(0,\infty)}\bigl\Vert 2v(s)-\Delta _{4}v(s)-\nabla_{4}v(s)-p_{4}(s)- \Delta_{2}v(s)-\nabla _{2}v(s)-p_{2}(s)\bigr\Vert , \\ &d(f_{5}v,f_{2}v) =\max_{s\in(0,\infty)}\bigl\Vert 2v(s)-\Delta _{5}v(s)-\nabla_{5}v(s)-p_{5}(s)- \Delta_{2}v(s)-\nabla _{2}v(s)-p_{2}(s)\bigr\Vert , \\ &d(f_{4}v,f_{1}u) =\max_{s\in(0,\infty)}\bigl\Vert 2v(s)-\Delta _{4}v(s)-\nabla_{4}v(s)-p_{4}(s)- \Delta_{1}u(s)-\nabla _{1}u(s)-p_{1}(s)\bigr\Vert , \\ &d(f_{5}v,f_{1}u) =\max_{s\in(0,\infty)}\bigl\Vert 2v(s)-\Delta _{5}v(s)-\nabla_{5}v(s)-p_{5}(s)- \Delta_{1}u(s)-\nabla _{1}u(s)-p_{1}(s)\bigr\Vert . \end{aligned} \right\} $$

From condition (2) of Theorem 2.1, we have

$$\begin{aligned}& \max\bigl\Vert \Delta_{1}u(s)+\nabla_{1}u(s) +p_{1}(s)- \Delta_{2}u(s)-\nabla _{2}u(s)-p_{2}(s)\bigr\Vert \\& \quad \leq\lambda\max \biggl\{ \frac{ \Upsilon_{2}\times\Upsilon_{3}\times\Upsilon_{4}\times\Upsilon _{5}+\Upsilon_{6}\times\Upsilon_{7}\times\Upsilon_{8}\times\Upsilon _{9}}{1+ (\max_{s\in(a,b)}\Upsilon_{4} )\cdot (\max_{s\in(a,b)}\Upsilon_{5} ) + (\max_{s\in(a,b)}\Upsilon_{8} )\cdot (\max_{s\in(a,b)}\Upsilon_{9} )} \biggr\} , \end{aligned}$$

which implies that

$$\begin{aligned}& \max_{s\in(0,\infty)} \bigl\Vert \Delta_{1}u(s)+\nabla _{1}u(s)+p_{1}(s)-\Delta_{2}u(s)- \nabla_{2}u(s)-p_{2}(s)\bigr\Vert \\& \quad \leq \lambda \biggl\{ \frac{ (\max_{s\in(0,\infty)}\Upsilon _{2} )\cdot (\max_{s\in(0,\infty)}\Upsilon_{3} )\cdot (\max_{s\in(0,\infty)}\Upsilon_{4} )\cdot (\max_{s\in(0,\infty)}\Upsilon_{5} )}{ 1+ (\max_{s\in(0,\infty)}\Upsilon_{4} )\cdot (\max_{s\in(0,\infty)}\Upsilon_{5} )+ (\max_{s\in(0,\infty)}\Upsilon_{8} )\cdot (\max_{s\in(0,\infty)}\Upsilon_{9} )} \\& \qquad {}+\frac{ (\max_{s\in(0,\infty)}\Upsilon _{6} )\cdot (\max_{s\in(0,\infty)}\Upsilon_{7} )\cdot (\max_{s\in(0,\infty)}\Upsilon_{8} )\cdot (\max_{s\in(0,\infty)}\Upsilon_{9} )}{ 1+ (\max_{s\in(0,\infty)}\Upsilon_{4} )\cdot (\max_{s\in(0,\infty)}\Upsilon_{5} ) + (\max_{s\in(0,\infty)}\Upsilon_{8} )\cdot (\max_{s\in(0,\infty)}\Upsilon_{9} )} \biggr\} , \end{aligned}$$

using (3.4), we get

$$\begin{aligned} d(f_{1}u,f_{2}v) \leq&\lambda \biggl\{ \frac {d(f_{3}u,f_{1}u)d(f_{6}u,f_{1}u)d(f_{3}u,f_{2}v)d(f_{6}u,f_{2}v)}{1+d(f_{3}u,f_{2}v)d(f_{6}u,f_{2}v)+d(f_{4}v,f_{1}u)d(f_{5}v,f_{1}u)} \\ &{}+\frac {d(f_{4}v,f_{2}v)d(f_{5}v,f_{2}v)d(f_{4}v,f_{1}u)d(f_{5}v,f_{1}u)}{1+d(f_{3}u,f_{2}v)d(f_{6}u,f_{2}v)+d(f_{4}v,f_{1}u)d(f_{5}v,f_{1}u)} \biggr\} . \end{aligned}$$

Now, to show that $f_{1}(Z) \subseteq f_{4}(Z)$, we have

$$\begin{aligned}& f_{4}\bigl(f_{1}u(s)+p_{4}(s)\bigr) \\& \quad = 2\bigl[f_{1}u(s)+p_{4}(s)\bigr]- \Delta_{4} \bigl(f_{1}u(s)+p_{4}(s)\bigr)- \nabla_{4}\bigl(f_{1}u(s)+p_{4}(s) \bigr)-p_{4}(s) \\& \quad = f_{1}u(s)+f_{1}u(s)+p_{4}(s)- \Delta_{4}\bigl(f_{1}u(s)+p_{4}(s) \bigr)- \nabla_{4}\bigl(f_{1}u(s)+p_{4}(s)\bigr) \\& \quad = f_{1}u(s)+\Delta_{1}u(s)+\nabla_{1}u(s)+p_{1}(s)+p_{4}(s) \\& \qquad {}- \Delta _{4}\bigl(\Delta_{1}u(s)+\nabla_{1}u(s)+p_{1}(s)+p_{4}(s) \bigr) \\& \qquad {}-\nabla_{4}\bigl(\Delta_{1}u(s)+\nabla _{1}u(s)+p_{1}(s)+p_{4}(s)\bigr). \end{aligned}$$

Using ($\mathrm{C}^{*}_{1}$), we get $f_{4} (f_{1}u(s)+p_{4}(s) )= f_{1}u(s)$, which implies that $f_{1}(Z) \subseteq f_{4}(Z)$. Similarly, one can prove that $f_{1}(Z)\subseteq f_{5}(Z) $, $f_{2}(Z)\subseteq f_{3}(Z)$ and $f_{2}(Z)\subseteq f_{6}(Z)$.

Next, we need to show the weak compatibility of the pair $(f_{1},f_{3})$. For this purpose,

$$\begin{aligned}& \bigl\Vert f_{3}f_{1}u(s)-f_{1}f_{3}u(s) \bigr\Vert \\& \quad = \bigl\Vert f_{3} \bigl(\Delta_{1}u(s)+ \nabla_{1}u(s)+p_{1}(s) \bigr)-f_{1} \bigl(2u(s)- \Delta_{3}u(s)-\nabla_{3}u(s)-p_{3}(s) \bigr) \bigr\Vert \\& \quad = \bigl\Vert 2 \bigl(\Delta_{1}u(s)+\nabla_{1}u(s)+p_{1}(s) \bigr)-\Delta _{3} \bigl(\Delta_{1}u(s)+ \nabla_{1}u(s)+p_{1}(s) \bigr) \\& \qquad {}-\nabla_{3} \bigl(\Delta_{1}u(s)+ \nabla_{1}u(s)+p_{1}(s) \bigr)-p_{3}(s)- \Delta_{1} \bigl(2u(s)-\Delta_{3}u(s)-\nabla _{3}u(s)-p_{3}(s) \bigr) \\& \qquad {}-\nabla_{1} \bigl(2u(s)-\Delta_{3}u(s)- \nabla_{3}u(s)-p_{3}(s) \bigr)-p_{1}(s) \bigr\Vert . \end{aligned}$$

If $f_{1}u(s)=f_{3}u(s)$, for $u(s)\in Z$. Then $\Delta_{1}u(s)+\nabla _{1}u(s)+p_{1}(s)=2u(s)-\Delta_{3}u(s)-\nabla_{3}u(s)-p_{3}(s)$, thus the above equation becomes

$$\begin{aligned}& \bigl\Vert f_{3}f_{1}u(s)-f_{1}f_{3}u(s) \bigr\Vert \\& \quad = \bigl\Vert 2\bigl(2u(s)-\Delta_{3}u(s)- \nabla_{3}u(s)-p_{3}(s) \bigr)-\Delta_{3} \bigl(2u(s)-\Delta_{3}u(s)-\nabla_{3}u(s)-p_{3}(s) \bigr) \\& \qquad {}-\nabla_{3}\bigl(2u(s)-\Delta_{3}u(s)- \nabla_{3}u(s)-p_{3}(s) \bigr)-p_{3}(s)- \Delta_{1}\bigl(\Delta_{1}u(s)+\nabla_{1}u(s)+p_{1}(s) \bigr) \\& \qquad {}-\nabla_{1}\bigl(\Delta_{1}u(s)+ \nabla_{1}u(s)+p_{1}(s) \bigr)-p_{1}(s)\bigr\Vert \\& \quad = \bigl\Vert 4u(s)-2\Delta_{3}u(s)-2\nabla_{3}u(s)-p_{1}(s)-3p_{3}(s) \\& \qquad {}-\Delta_{1}\bigl(\Delta_{1}u(s)+ \nabla_{1}u(s)+p_{1}(s)\bigr)-\nabla _{1}\bigl( \Delta_{1}u(s)+\nabla_{1}u(s)+p_{1}(s)\bigr) \\& \qquad {}-\Delta_{3}\bigl(2u(s)-\Delta_{3}u(s)- \nabla_{3}u(s)-p_{3}(s) \bigr) \\& \qquad {}-\nabla_{3} \bigl(2u(s)-\Delta_{3}u(s)-\nabla_{3}u(s)-p_{3}(s) \bigr)\bigr\Vert , \end{aligned}$$

with the help of ($\mathrm{C}^{*}_{3}$), we get $\|f_{3}f_{1}z(s)-f_{1}f_{3}z(s)\| =0$, which implies that $f_{3}f_{1}z(s)=f_{1}f_{3}z(s)$, whenever $f_{1}z(s)=f_{3}z(s)$. Thus the pair $(f_{1},f_{3})$ is weakly compatible. In a similar way one can easily show the weakly compatibility of the pairs $(f_{2},f_{4})$, $(f_{1},f_{6})$, and $(f_{2},f_{5})$. Also, from condition (1) of Theorem 3.3 the pairs $(f_{2},f_{4})$ and $(f_{2},f_{5})$ satisfy the common $(CLR_{f_{2}})$-property. Thus by Corollary 3.1, we can find a unique common fixed point of $f_{1}$, $f_{2}$, $f_{3}$, $f_{4}$, $f_{5}$, and $f_{6}$ in *Z*, that is, the system (3.1) of Volterra-Hammerstein non-linear integral equations has a unique common solution in *Z*. □

In the next theorem we use the common $(E.A)$-property.

### Theorem 3.4

*Under the assumptions* ($\mathrm{C}_{1}^{*}$)-($\mathrm{C}_{4}^{*}$) *and the conditions* (2), (3) *of Theorem* 3.3, *if there exist two sequences* $\{{z_{n}}\}$ *and* $\{{w_{n}}\}$ *in* *Z* *such that*

3.5 $$ \lim_{n\rightarrow\infty}f_{2}{z_{n}}=\lim _{n\rightarrow\infty }f_{4}{z_{n}}=\lim _{n\rightarrow\infty}f_{2}{w_{n}}=\lim _{n\rightarrow\infty }f_{5}{w_{n}}=z, \quad \textit{for some } z\in Z, $$

*and both* $f_{4}(Z)$ *and* $f_{5}(Z)$ *are closed subspaces of* *Z*, *then the system* (3.1) *of Volterra*-*Hammerstein equations has a unique common solution*.

## Conclusions

In the current work, we studied the existence of unique common solution for the systems of Urysohn and Volterra-Hammerstein integral equations in incomplete spaces. Several problems that appear in applied mathematics, physical sciences, geology, mechanics, engineering, economics, and biology generate mathematical models described by integral equations.
